# A comparative map of macroautophagy and mitophagy in the vertebrate eye

**DOI:** 10.1080/15548627.2019.1580509

**Published:** 2019-02-20

**Authors:** Thomas G. McWilliams, Alan R. Prescott, Beatriz Villarejo-Zori, Graeme Ball, Patricia Boya, Ian G. Ganley

**Affiliations:** a MRC Protein Phosphorylation and Ubiquitylation Unit, Sir James Black Centre School of Life Sciences, University of Dundee, Dundee, UK; b Translational Stem Cell Biology & Metabolism Program, Research Programs Unit, Faculty of Medicine, University of Helsinki, Finland; c Dundee Imaging Facility, School of Life Sciences, University of Dundee, Dundee, UK; d Department of Cellular and Molecular Biology, Centro de Investigaciones Biológicas, CSIC, Madrid, Spain

**Keywords:** Autophagy, ciliary body, cornea, eye, hyaloid, lens, mitochondria, *mito*-QC, mitophagy, retina

## Abstract

Photoreception is pivotal to our experience and perception of the natural world; hence the eye is of prime importance for most vertebrate animals to sense light. Central to visual health is mitochondrial homeostasis, and the selective autophagic turnover of mitochondria (mitophagy) is predicted to play a key role here. Despite studies that link aberrant mitophagy to ocular dysfunction, little is known about the prevalence of basal mitophagy, or its relationship to general autophagy, in the visual system. In this study, we utilize the *mito*-QC mouse and a closely related general macroautophagy reporter model to profile basal mitophagy and macroautophagy in the adult and developing eye. We report that ocular macroautophagy is widespread, but surprisingly mitophagy does not always follow the same pattern of occurrence. We observe low levels of mitophagy in the lens and ciliary body, in stark contrast to the high levels of general MAP1LC3-dependent macroautophagy in these regions. We uncover a striking reversal of this process in the adult retina, where mitophagy accounts for a larger degree of the macroautophagy taking place, specifically in the photoreceptor neurons of the outer nuclear layer. We also show the developmental regulation of autophagy in a variety of ocular tissues. In particular, mitophagy in the adult mouse retina is reversed in localization during the latter stages of development. Our work thus defines the landscape of mitochondrial homeostasis in the mammalian eye, and in doing so highlights the selective nature of autophagy *in vivo* and the specificity of the reporters used.

**Abbreviations:** ATG: autophagy related; GFP: green fluorescent protein; LC3: microtubule associated protein 1 light chain 3; ONH: optic nerve head; ONL: outer nuclear layer; RPE: retinal pigment epithelium.

## Introduction

Although general macroautophagy has been studied extensively in the context of nutrient deprivation, it has emerged that several types of autophagy exist to orchestrate cellular homeostasis. These include selective pathways that can degrade damaged or superfluous organelles *via* autophagy. Mitochondrial autophagy (mitophagy) is emerging as a key pathway in the regulation of mitochondrial network integrity [1–3]. Although the great majority of research has focused on mitophagy as an induced cellular stress-response to mitotoxic damage, recent studies describing mitophagy reporter mice have revealed the pervasive and basal nature of mammalian mitophagy *in vivo* [1,2]. Mitochondrial homeostasis is also critical for ocular health [4]. This is strongly evidenced by a variety of ophthalmic manifestations in both rare and common conditions, including inherited mitochondrial diseases, diabetic retinopathy and glaucoma, respectively [5–12]. Furthermore, disrupted autophagic signalling has been reported in a variety of ocular disease contexts (reviewed in [4]). Although macroautophagy has been studied in the mammalian eye since the late 1970’s [13], we still do not understand the extent of selective autophagy here. This is principally due to a lack of sensitive tools that enable comparative analysis of selective versus non-selective autophagy *in vivo*. Given the allure of dysregulated mitophagy as a contributor to a variety of disease states, it is important to understand the normal mitochondrial landscape of tissue and its mitophagic regulation *in vivo*.

Comprised of several unique tissues, the mammalian eye can be broadly classified into three distinct regions: the corneo-scleral, uveal and retinal layers. In this resource we focus on these regions, in particular on selected ocular structures of major clinical interest, where several studies have inferred the involvement of macroautophagy. Light enters the eye *via* the cornea, a refractive, transparent and avascular structure that focuses light on the retina. Defective autophagic flux is associated with genetic and damage-induced corneal pathology [14–16]. Posterior to the cornea lies the lens, another transparent and avascular tissue consisting of a unique tapestry of epithelial and fiber cells. In concert with the cornea, the lens functions to focus light on the retina [17,18]. Lens fiber cells are elongated structures that undergo a striking elimination of all membrane bound organelles as they mature. Although the consensus agrees the importance of autophagy as a cellular quality control mechanism in the developing and mature lens, its precise contribution to organelle elimination here remains unclear [4,17]. The retina is a constituent of the central nervous system, and a vascularised region of high-metabolic demand. Multiple studies have demonstrated the metabolic significance of general autophagy in the retina, and mitophagy is predicted to be particularly important regulator of retinal viability in health and disease [4].

Owing to the difficulty of studying the selective turnover of mitochondria in mammalian tissues, there has been an understandable lack of data on mitophagy in the retina and associated tissues of the eye. However, using our *mito*-QC mouse model [19], we have now been able to characterize mitophagy and mitochondrial network morphology in the adult and developing eye. Recently, we described the phenomenon of retinal mitophagy using *mito-*QC [20] and in the present study, we expand upon this initial description by characterizing the spatio-temporal regulation of mitochondrial homeostasis in the eye as a whole. We further extend this work by comparing the degree of mitophagy with that of general macroautophagy, by using a recently described autophagy reporter mouse based on the same principle, and generated in the same way, as *mito*-QC [19,20]. Surprisingly, high levels of mitophagy and general macroautophagy do not always coincide, highlighting the specificity of our reporter systems and demonstrating the selective nature of autophagy *in vivo*. Given the postulated contribution of mitochondrial dysfunction to many ophthalmological disorders, this resource should be of broad interest to vision researchers with an interest in mitochondrial biology, in addition to those studying the physiological implications of autophagy.

## Results

In this Resource we follow the path of light through the eye and describe macroautophagy from the cornea to the optic nerve in adult and embryonic day 16.5 (E16.5) eyes. To reveal the nature and degree of macroautophagy in the eye, we utilized our previously characterized and validated mouse models that monitor mitophagy [19] and total macroautophagy [20]. Both models rely on similar transgenes expressed from the *Rosa26* locus that utilize a tandem mCherry-GFP tag, either attached to the mitochondria through the outer membrane targeting region of FIS1 (amino acids 101-152); or the autophagosome itself through attachment to the N terminus of MAP1LC3B/LC3B (microtubule associated protein 1 light chain 3 beta). In these instances, when a mitochondrion or autophagosome undergoes lysosomal delivery, the acidic microenvironment is sufficient to quench the GFP signal, but not that from mCherry. Hence the degree of mitophagy or general macroautophagy can be determined from the appearance and number of mCherry-only foci (mito/autolysosomes) in tissue sections. The almost identical nature of these mouse models, combined with the fact that they were generated and bred in the same way, gives us a unique opportunity to determine the relative degree of mitophagy, with respect to general macroautophagy, occurring *in vivo*.

### Comparative analysis of autophagy and mitophagy in the cornea

**Figure 1.**
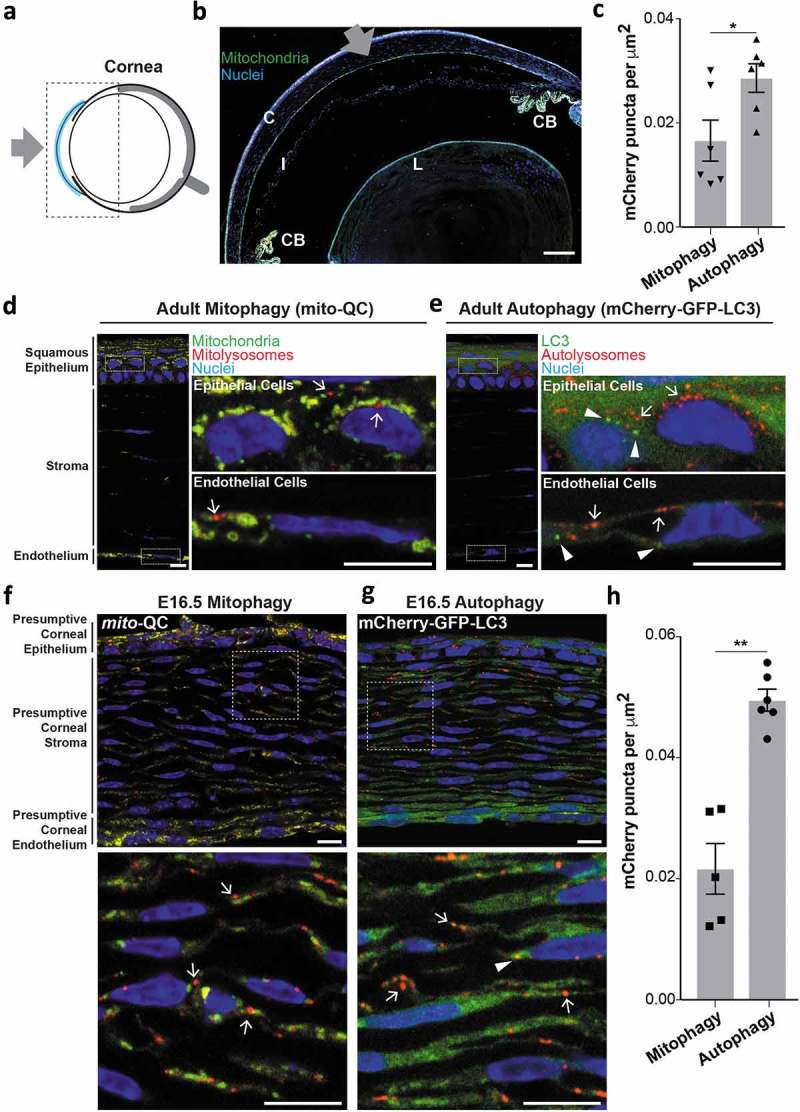
Basal mitophagy and macroautophagy in the cornea. (a) Schematic of the mouse eye depicting location of cornea in blue. Boxed area shown in panel (b). Gray arrow represents the light path i.e. direction of light entering the eye. (b) Overview micrograph of *mito*-QC eye section depicting cornea (C), iris (I), ciliary body (CB) and lens (L). Gray arrow represents light path. Scale bar: 100 *μ*m. (c) Quantification of mCherry-only puncta from *mito*-QC (mitophagy) or mCherry-GFP-LC3 (autophagy) corneal eye sections. Data points are means from individual animals ±SEM (*P < 0.05 (0.0342)). (d) Representative *mito*-QC optical section showing mitophagy in the cornea. Boxed regions are shown magnified on the right and arrows mark instances of mitophagy defined by the presence of mCherry-only mitolysosomes. (e) Representative mCherry-GFP-LC3 section showing autophagy in the cornea. Boxed regions are shown magnified on the right and arrows mark examples of autolysosomes and arrowheads indicate presumptive autophagosomes. (f) Optical section detailing pronounced mitophagy in the *mito*-QC E16.5 corneal stroma. Boxed region is shown magnified below with arrows marking examples of mitolysosomes. (g) Optical section detailing autophagy in the mCherry-GFP-LC3 E16.5 corneal stroma. Boxed region is shown magnified below with autolysosomes (arrows) autophagosomes (arrowheads). (h) Quantification of mCherry-only puncta from *mito*-QC (mitophagy) or mCherry-GFP-LC3 (autophagy) E16.5 corneal eye sections. Data points are means from individual animals ±SEM (**P < 0.01 (0.0012)). Scale bars (panels d-g): 10 *μ*m.

Often referred to as the ‘window of the eye’ the cornea is a transparent, avascular structure and accounts for 80% of the refractive power of the eye. Its fixed surface convexity enables images to be focused on the retina and an overview of the cornea is shown in Figure 1(a,b). The cornea consists of a stratified multicellular epithelium, a multi-lamellar collagenous stroma interlaced with keratocytes, a proteinaceous acellular layer (Descemet’s membrane) and a monolayer of inter-digitated secretory endothelial cells. Its anterior location means the cornea directly interfaces with the immediate environment, where it is exposed to myriad environmental stresses and serves as a protective barrier by absorbing UV-B light. Impaired autophagic flux and mitochondrial quality control in the cornea has been linked to granular corneal dystrophy type 2, Fuchs’ endothelial corneal dystrophy and non-nephropathic cystinosis [21–23]. Furthermore, impaired mitochondrial morphology and defective respiration has been observed in human corneal biopsies from diabetic patients [24]. Metabolic and classical electron microscopy-based studies have established the cornea as mitochondrially rich, and using eye cryosections from adult *mito*-QC mice, we were able to verify this (Figure 1(d)). Mitochondria in the epithelial layer appeared punctate and highly ordered, yet not as networked as those in the endothelial layer. Mitophagy, as evidenced by the appearance of mCherry-only puncta, was also apparent in both the epithelial and endothelial layers (Figure 1(c,d)). In contrast to mitophagy, there was a greater abundance of general autophagy as visualised in cryosections from mCherry-GFP-LC3 adult mice (Figure 1(c,e)). On comparing the 2 models, it would thus appear that mitophagy accounts for only a fraction of the total corneal macroautophagy. A strict quantitative comparison is challenging given that the dynamics of autophagosome fusion with lysosomes, as well as the relative reporter labelling of mitochondria *vs*. autophagosomes, would also need to be considered.

As *in vivo* mitophagy is a developmentally-regulated process [19] and in particular is important for retinal development [25], we next explored if this phenomenon occurs in the developing cornea. We thus examined the cornea in eye sections from embryonic *mito*-QC and mCherry-GFP-LC3 animals in more detail at E16.5. We observed a slight increase in corneal mitophagy compared to adult, which is likely accounted for by the increased number of developing stromal keratinocytes in the presumptive stromal region (Figure 1(f,h)). In contrast, corneal autophagy in general at E16.5 is increased by 60% compared to the adult cornea (Figure 1(g,h)). This period in development is coincident with the synthesis of many matrix molecules in the endothelium, and may represent the discovery of an unknown stage of metabolic adaption or remodelling in the mammalian eye [26]. Regardless, the differential changes in mitophagy versus general autophagy demonstrate the selective nature of mammalian autophagy *in vivo* and highlight the specificity of the reporters.

### Mitophagy in the lens

**Figure 2.**
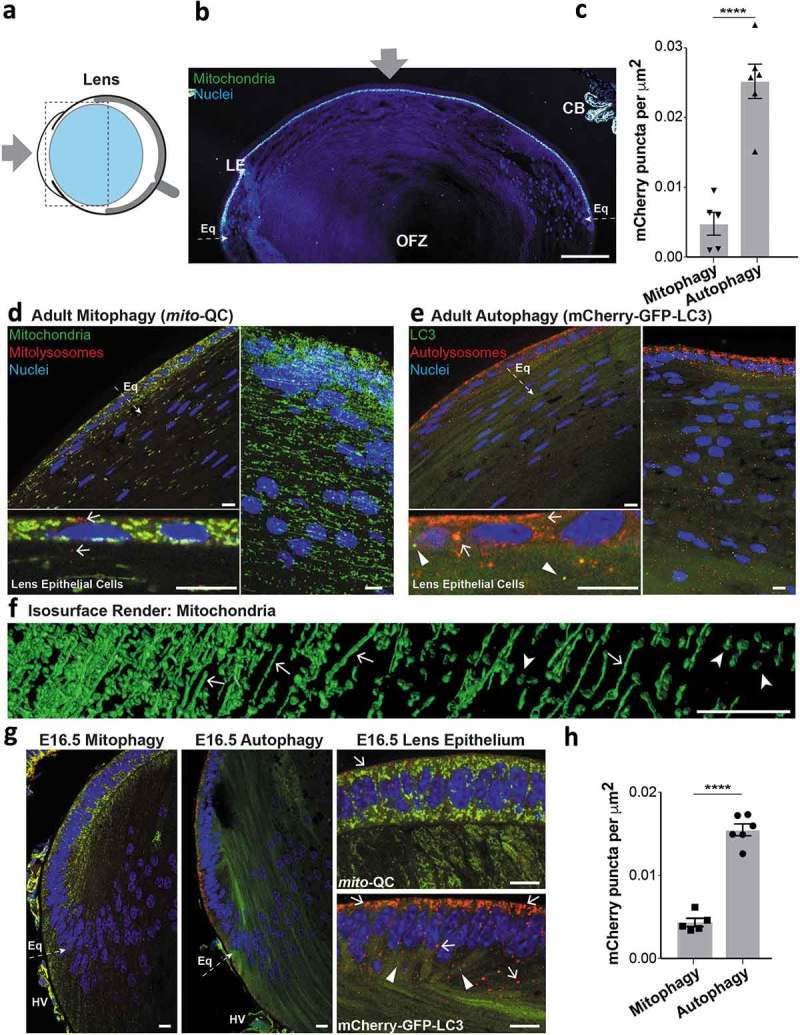
Mitochondrial architecture, mitophagy and autophagy in the adult lens. (a) Schematic of the mouse eye depicting anatomical location of the lens in light blue. Boxed area is shown in panel (b) and gray arrow represents light path. (b) Overview photomicrograph of *mito*-QC eye section depicting the lens; LE, lens epithelium; Eq, equatorial region; OFZ, organelle-free zone; CB, ciliary body. Gray arrow, as in panel A. Scale bar: 100 *μ*m. (c) Quantification of mCherry-only puncta from *mito*-QC (mitophagy) or mCherry-GFP-LC3 (autophagy) lens sections. Data points represent means from individual animals ±SEM (***P < 0.0001). (d) Upper left panel shows optical section of the adult lens equatorial region from a *mito*-QC mouse, below is magnified area of lens epithelium showing small spherical mitochondria and minimal mitophagy, with examples of mitolysosomes marked by arrows. Panel on right shows maximum Z-projection of lens optical slices. (e) Upper left panel shows optical section of the adult lens equatorial region from a mCherry-GFP-LC3 mouse, below is magnified area of lens epithelium with examples of autolysosomes marked by arrows and autophagosomes by arrowheads. Panel on right shows Z-projection of lens optical slices. (f) Isosurface render detailing mitochondrial network in *mito*-QC with lens epithelium on the left side of the panel (lateral to medial = left to right). Arrows denote examples of elongated mitochondria and arrowheads that of smaller solitary mitochondria. (g) Optical sections from E16.5 eyes detailing equatorial region (Eq), hyaloid vessels (HV) are also marked. Magnified epithelium sections are shown on the right, with arrows showing examples of mitolysosomes (top panel) and autolysosomes (bottom panel). Arrowheads mark autophagosomes. (h) Quantification of mCherry-only puncta from *mito*-QC (mitophagy) or mCherry-GFP-LC3 (autophagy) E16.5 lens sections. Data points represent means from individual animals ±SEM (***P < 0.0001). Scale bars (panels D-G): 10 *μ*m.

The lens is a biconvex, avascular and translucent structure: thousands of anucleated fibers stretch between its anterior and posterior poles in a precise geometrical arrangement [27]. This unique form of the lens enables its function by facilitating the focus of light on the retina. Each fiber has the shape of a 6-sided prism and is highly enriched in crystallin proteins [17,28]. Lens fibers are tightly packed and enveloped by the lens capsule, connected by a suspensory ligament to the ciliary body. The anterior lens is covered by a cuboidal epithelium of nucleated cells, which merge with a proliferative layer at the equatorial margin (Figure 2(a,b)). Damage to lens epithelial or fiber cells (e.g. ionising radiation) or disrupted lens development can induce opacities that cause cataracts, estimated to account for 50% of global blindness [29]. Of relevance here, ATG5-dependent macroautophagy is essential to prevent cataractogenesis [17]. Despite extensive research on autophagy in the adult lens, little is known about mitochondrial network homeostasis in this structure. Interestingly, cryosections from adult *mito*-QC mice revealed little evidence of mitophagy in the adult lens fiber cells or in the lens epithelium (Figure 2(c,d)). The *mito*-QC signal was also sufficient to resolve the morphology of mitochondria in lens equatorial fibers (Figure 2(d,f)). 3D volume rendering of the equatorial region revealed highly elongated mitochondria, interspersed with smaller circular mitochondria, with the total mitochondrial density decreasing towards the central organelle-free zone (Figure 2(f)). Mitochondria of the lens epithelium were noticeably different in morphology, appearing more punctate and less networked than those in adjacent fiber cells (Figure 2(d)). The lack of mitophagy in the adult mouse lens contrasts with published descriptions of mitophagy and autophagy in the adult human lens [18]. However, as these studies relied upon electron microscopy, it is somewhat difficult to discern differences between mitophagy specifically over general autophagy. Consistent with this, cryosections from adult autophagy reporter mice showed pronounced autophagy in the lens epithelium, and to a lesser extent in fiber cells (Figure 2(c,e)). These data demonstrate that although LC3-dependent macroautophagy occured to a high degree in lens, the selective elimination of mitochondria by mitophagy proceeded at a much lower level. This scenario also appears to manifest during lens embryonic development at E16.5, with a low level of mitophagy compared to general macroautophagy in the lens epithelium (Figure 2(g,h)). Our work supports previously published data suggesting that organelle clearance during lens fiber development does not proceed *via* macroautophagy [30].

In contrast, we did observe a high degree of mitophagy in the associated hyaloid vasculature proximal to the developing lens (Figure S1). Blood supply in the developing eye is sustained *in utero* by the formation of a transient intraocular circulatory system, known as the hyaloid vasculature. This dense vascular network irrigates the developing eye prior to the formation of the mature intraretinal vascular network [31]. The hyaloid network is characterised by the absence of a venous system, thus all hyaloid vessels are regarded as arterial in nature. The hyaloid network breaks down or regresses during mammalian development (postnatal period in mice, and during month 5 of human gestation – second trimester). Both WNT7B (Wnt family member 7B) paracrine signalling by macrophages and KDR/VEGFR2 (kinase insert domain receptor)-signalling by retinal neurons are known to contribute to hyaloid vessel regression in mammals [32–34]. The death of the hyaloid network correlates with the reciprocal maturation of the retina and lens, upon which the homeostatic support of the retina becomes fulfilled by its developing vascular network. Although general autophagy has been reported in this structure and previously suggested to regulate hyaloid vessel regression during development [35], the occurrence of selective autophagy in this structure remains unclear. In sections of E16.5 eyes, we observed pronounced autophagy and specifically mitophagy in hyaloid vasculature, likely at the beginning stages of their regression (Figure S1B). Using volume imaging and 3D rendering software, we were also able to profile mitochondrial morphology and mitophagy in these structures (Figure S1C and D). To our knowledge, this is the first demonstration of *in vivo* mitophagy that precedes a defined phase of vertebrate developmental apoptosis in mammals. Future studies will be essential to determine the physiological relevance of mitophagy pre-empting developmental apoptosis in this tissue, by a more systematic investigation during actual hyaloid regression. It is of note that we did not detect the presence of cleaved caspase 3, a marker of apoptosis, at this or neonatal stages (results not shown). However, hyaloid regression is known to be a complex process, driven by paracrine WNT7B macrophage-dependent clearance [32,33], with a contribution from KDR-dependent signalling by retinal neurons [34]. Our demonstration of mitophagy here is the first evidence for selective autophagy in this tissue *in vivo*. Incomplete hyaloid regression is associated with severe ocular pathology in humans, called persistent hyperplastic primary vitreous that manifests with severe intraocular haemorrhage, retinal detachment and cataract [36]. It will be interesting to assess if defective mitophagy plays a role in these diseases.

### Mitophagy in the ciliary body

**Figure 3.**
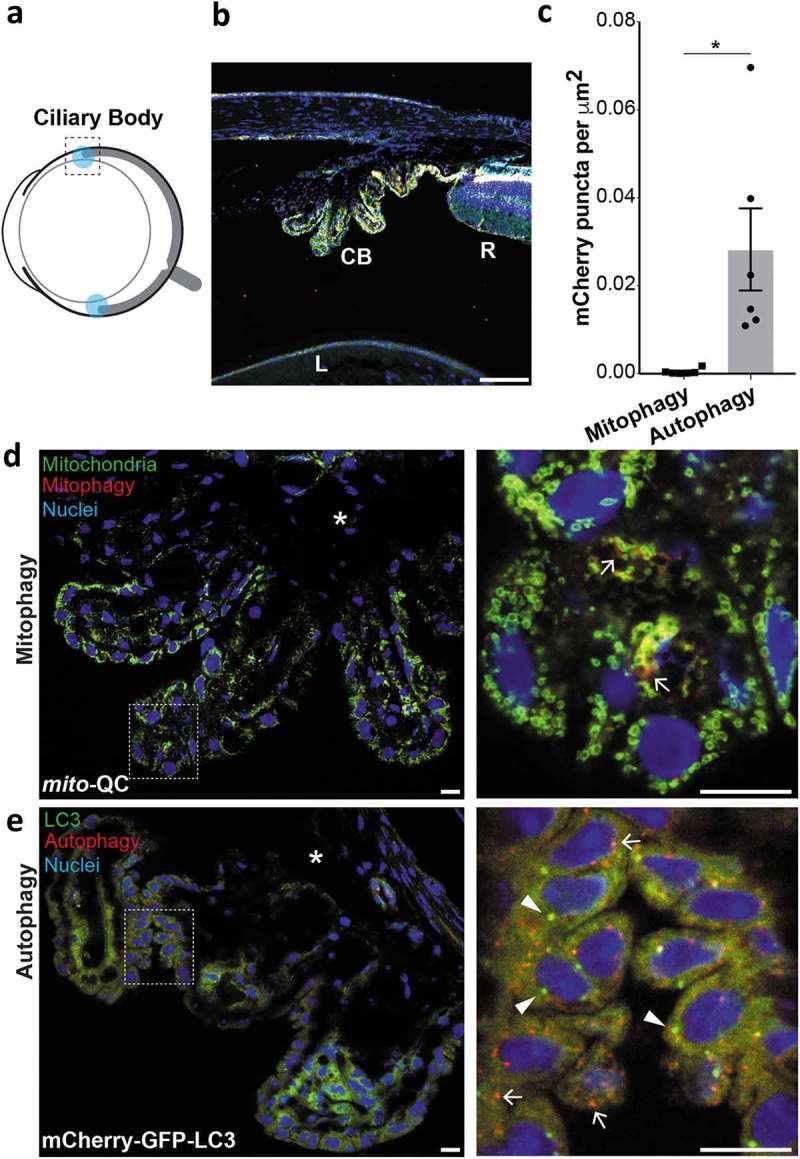
Mitophagy and macroautophagy in the ciliary body. (a) Schematic of the adult mouse eye depicting anatomical location of the ciliary bodies in light blue. (b) Optical section from *mito*-QC eye showing ciliary body (CB); peripheral retina (R); lens (L). Scale bar: 100 *μ*m. (c) Quantification of mCherry-only puncta from *mito*-QC (mitophagy) or mCherry-GFP-LC3 (autophagy) ciliary body sections. Data points represent means from individual animals ±SEM (*P < 0.05 (0.031)). (d) Optical section from *mito*-QC eye detailing ciliary body. Boxed region is shown magnified on the right and arrows mark examples of mitolysosomes. Asterisk marks area of highly pigmented ciliary epithelium, with the pigment reducing the mCherry-GFP fluorescence. (e) Optical section from mCherry-GFP-LC3 eye detailing autophagy in the ciliary body. Boxed region is shown magnified on the right and arrows mark examples of autolysosomes and arrowheads autophagosomes. Asterisk marks area as in panel D. Scale bars: 10 *μ*m.

The unique spherical nature of the mammalian eye is governed by the existence of intraocular hydrostatic pressure [37]. Such pressure sustains the spherical integrity of the eye, and arises from the continual secretion of transparent aqueous humour produced by the ciliary body into the interior cavity. This flow facilitates metabolite exchange between cells of the cornea and lens. The ciliary body is highly vascularised with a considerable surface area for fluid secretion, owing to its unique topology of ciliary ridges covered by a ciliary epithelium [37], and its anatomy is well described ([38] and Figure 3(a,b)). Autophagy has been previously described in the ciliary body, yet mitochondrial biology here remains unexplored [39]. We found that mitochondria in the ciliary body appeared small, spherical and tightly compacted (Figure 3(d)). Surprisingly, we observed almost no mitophagy in these cells, contrasting to the much higher rate of general macroautophagy where autophagosomes and autolysosomes are abundant (Figure 3(c–e)). These observations suggest that basal autophagy may be an important regulator of ciliary body homeostasis and its dysfunction may be detrimental to cellular health. The most common ocular cancer in adults, uveal melanoma, can arise in the ciliary body [40], but the exact role autophagy plays here remains to be determined [41].

### Retinal mitophagy

**Figure 4.**
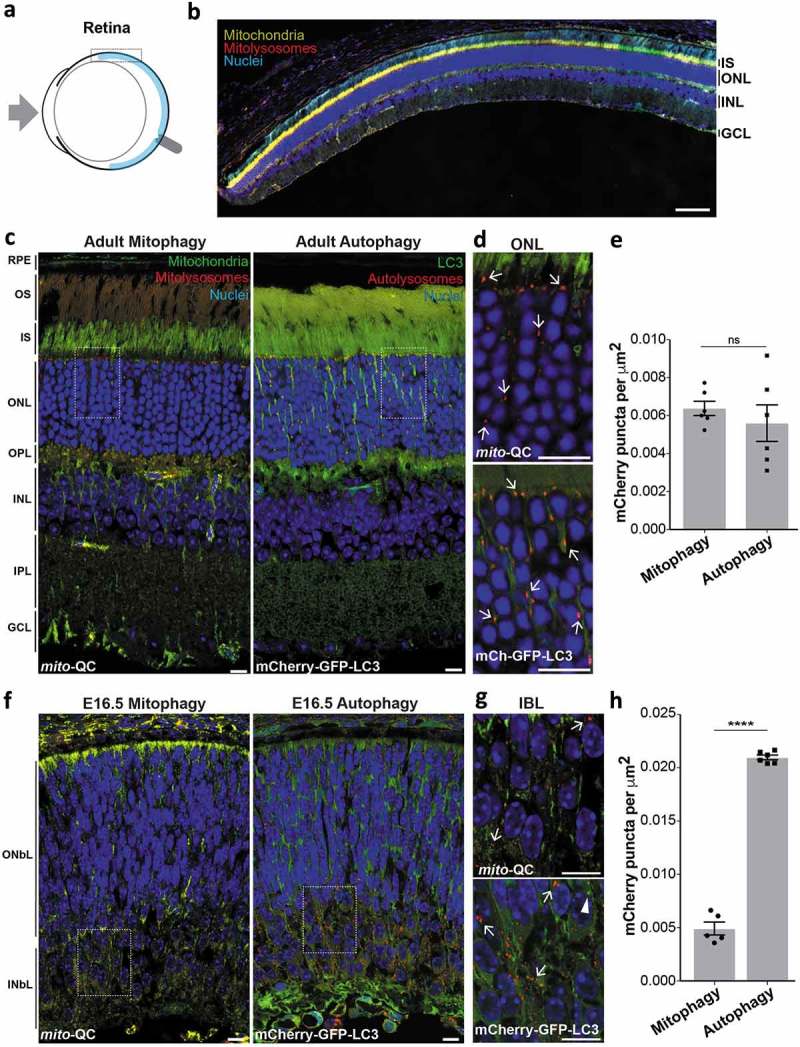
Mitophagy is highly localized to retinal photoreceptors. (a) Schematic of the mouse eye depicting anatomical location of the retina in light blue. Boxed region is shown in b. (b) Optical section from *mito*-QC retina: IS, inner segment; ONL, outer nuclear layer; INL; inner nuclear layer; GCL, ganglion cell layer. Scale bar: 100 *μ*m. (c) Optical sections from mito-QC and mCherry-GFP-LC3 retina. Boxed regions are shown in panel D. RPE, retinal pigment epithelium; OS, outer segment; OPL, outer plexiform layer; IPL, inner plexiform layer. (d) Magnified section of ONL from *mito*-QC (top panel) or mCherry-GFP-LC3 adult retina (bottom panel). Arrows mark examples of mitophagosomes or autolysosomes. (e) Quantification of mCherry-only puncta from *mito*-QC (mitophagy) or mCherry-GFP-LC3 (autophagy) retinal sections, including all the layers shown in panel C. Data points represent means from individual animals ±SEM (ns = *P > *0.05 (0.4772)). (f) Optical sections from *mito*-QC and mCherry-GFP-LC3 E16.5 retina. Boxed regions are shown in panel G. ONbL, outer neuroblast layer; INbL, inner neuroblast layer. (g) Magnified section of INbL from *mito*-QC (top panel) or mCherry-GFP-LC3 E16.5 retina (bottom panel). Arrows mark examples of mitophagosomes or autolysosomes and arrowhead indicates an autophagosome. (h) Quantification of mCherry-only puncta from *mito*-QC (mitophagy) or mCherry-GFP-LC3 (autophagy) E16.5 retinal sections. Data points represent means from individual animals ±SEM (***P < 0.0001). Scale bars (c-g): 10 *μ*m.

The retina is a constituent of the central nervous system and regarded as one of the most metabolically active mammalian tissues [42,43] (Figure 4(a,b)). Its multi-layered structure facilitates photoreception and the conversion of light energy to neural information that is transduced *via* the optic nerve. For over 40 years, autophagy has been studied in the retina and has been implicated in its development, maintenance and degeneration [4]. Indeed, *Atg5*-dependent autophagy is essential for photoreceptor viability [44–47]. Furthermore, aberrant mitochondrial dysfunction has been suggested to contribute to retinal pathology [48,49]. In addition, autophagy is tightly regulated in the retina and follows circadian variation [50,51]. Moreover, dysfunctional autophagy is observed in several models of retinal dystrophies [52]. Thus, elucidating how this process is regulated here constitutes an important research area of translational significance. As mitochondria lie at the heart of metabolism, it is essential to understand how mitochondrial quality control is regulated in the retina. Though less work has been carried out in terms of retinal mitophagy, early electron microscopy studies clearly depicted evidence for mitochondria in autophagosomal structures, suggesting it is a relevant process [13,50]. Consistent with this, we previously demonstrated a striking enrichment of mitophagy in the retinal outer nuclear layer (ONL) [20]. To ascertain the degree of mitophagy compared to LC3-dependent macroautophagy, we compared retinal sections from *mito*-QC and mCherry-GFP-LC3 mice. Surprisingly, we observed a pronounced and very similar enrichment of both mitolysosomes and autolysosomes in the ONL of *mito*-QC and autophagy-reporter mice respectively (Figure 4(c–e)). This indicates that a significant amount of macroautophagy taking place in the ONL can be attributed to mitophagy, which makes this area by far the highest degree of mitophagy (relative to total macroautophagy) occurring in the eye. Notably, we cannot say this is exclusively mitophagy as lysosomal delivery of other autophagic cargo in conjunction with mitochondria may occur.

To determine if retinal mitophagy is established during development we analyzed sections from E16.5 *mito*-QC eyes. In stark contrast to the adult eye, mitophagy at E16.5 appeared to be concentrated at a different level in the retina and was restricted to the inner neuroblast layer (Figure 4(f,g)). These data are consistent with recent findings that implicate programmed mitophagy as a developmental requirement of retinal ganglion cells, which are present within this region at this stage [25,53]. Also, in agreement with this study [25], there was much more general autophagy in the developing retina (Figure 4(f–h)). The level of macroautophagy was greater in the inner neuroblast layer, however unlike mitophagy, autolysosomes were also present throughout the retina including the outer neuroblast layer. Our *in vivo* data provide compelling support for the growing body of evidence that underscores the physiological significance of mitophagy and macroautophagy in mammalian retinal development.

**Figure 5.**
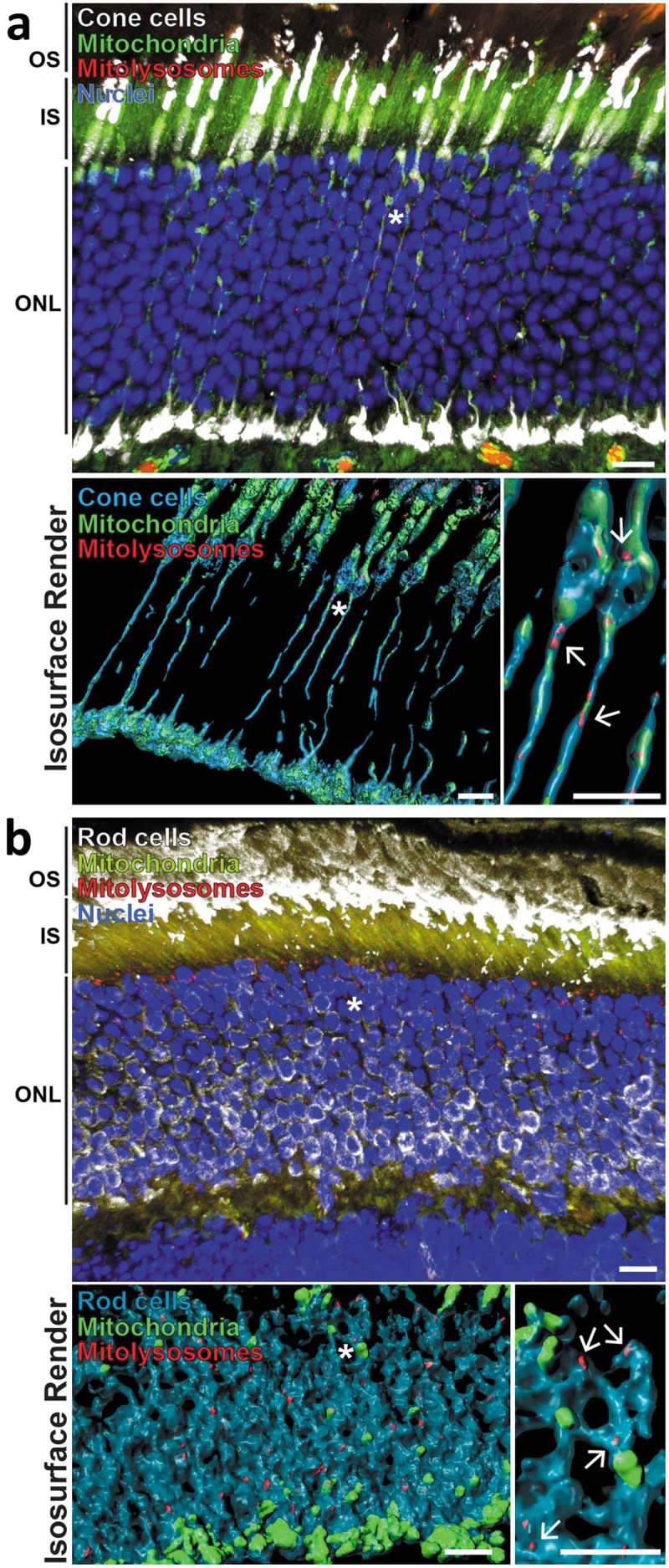
Mitophagy occurs in retinal photoreceptor neurons. (a) Z-projection showing adult *mito*-QC retinal outer nuclear layer (ONL) stained with antibodies against ARR3/cone arrestin (white, top panel). Inner segments (IS) and outer segments (OS) of the photoreceptor cells are also indicated. Lower left panel shows isosurface render of cone cells (cyan) from the above micrograph. Asterisk denotes cone projections magnified in lower right panel, arrows indicate mitolysosomes. Note presence of mitolysosomes in cone processes. (b) Z-projection of *mito*-QC retinal outer nuclear layer (ONL) and inner and outer photoreceptor cell segments (IS and OS respectively) stained with antibodies against SAG/visual arrestin to identify rod cells (white, top panel). Lower left panel shows isosurface render of rod somata (cyan) from the above micrograph. Asterisk denotes area of cells shown magnified in lower right panel and arrows mark mitolysosomes. Scale bars: 10 *μ*m.

Given the high degree of mitophagy present in the adult retinal ONL, we decided to investigate this further. The ONL contains the cell bodies of the photoreceptor neurons, with rod cells being the dominant type compared to cone cells (97% *vs* 3% respectively in mice [54]). To determine if mitophagy is restricted to one of these cell types we immunolabelled *mito*-QC cryosections using either anti-ARR3 (arrestin 3, retinal) antibodies to detect cones or anti-SAG/s-antigen visual arrestin antibodies to detect rods (Figure 5). ARR3 staining allowed visualization of entire cone cells including outer and inner segments as well as somata, axons and their termini. Importantly, mCherry-only puncta colocalized with ARR3, indicating that cone photoreceptors undergo mitophagy (Figure 5(a)). Isosurface rendering allowed us to visualise the spatial nature of mitophagy in these cells and showed that mitophagy is largely restricted to the soma, as we have seen in other neuronal cell types [19,20]. However, we did observe a higher frequency of axonal mitophagy compared to other regions of the CNS that we have previously published (Figure 5(a) – lower panels). As expected, staining with anti-SAG to mark rods revealed that this cell type is present in in much larger numbers (Figure 5(b)) and we were clearly able to identify significant numbers of mitolysosomes present here (Figure 5(b), lower panels).

Mitophagy appears to be highly enriched in certain neuronal populations such as Purkinje cells [19] or dopaminergic neurons [20], as well as the photoreceptors shown here. The rich diversity of neuronal subtypes and connections in the retina facilitates parallel information processing. Bipolar and retinal amacrine cells transmit signals from the photoreceptor neurons to the retinal ganglion cells, which in turn transmit this information to the visual cortex *via* the optic nerve. The restrictive nature of mitophagy to the ONL suggests that these neurons do not undergo significant mitophagy and this was confirmed to be the case through immunolabelling of these retinal cells (Figure S2). It is not clear why these neuronal subtypes are markedly different in their rates of basal mitophagy to others, and future work will be vital to determine the cell-specific regulation of this process in the retina. It will be particularly interesting to determine if mitophagy is related to spectral sensitivity.

**Figure 6.**
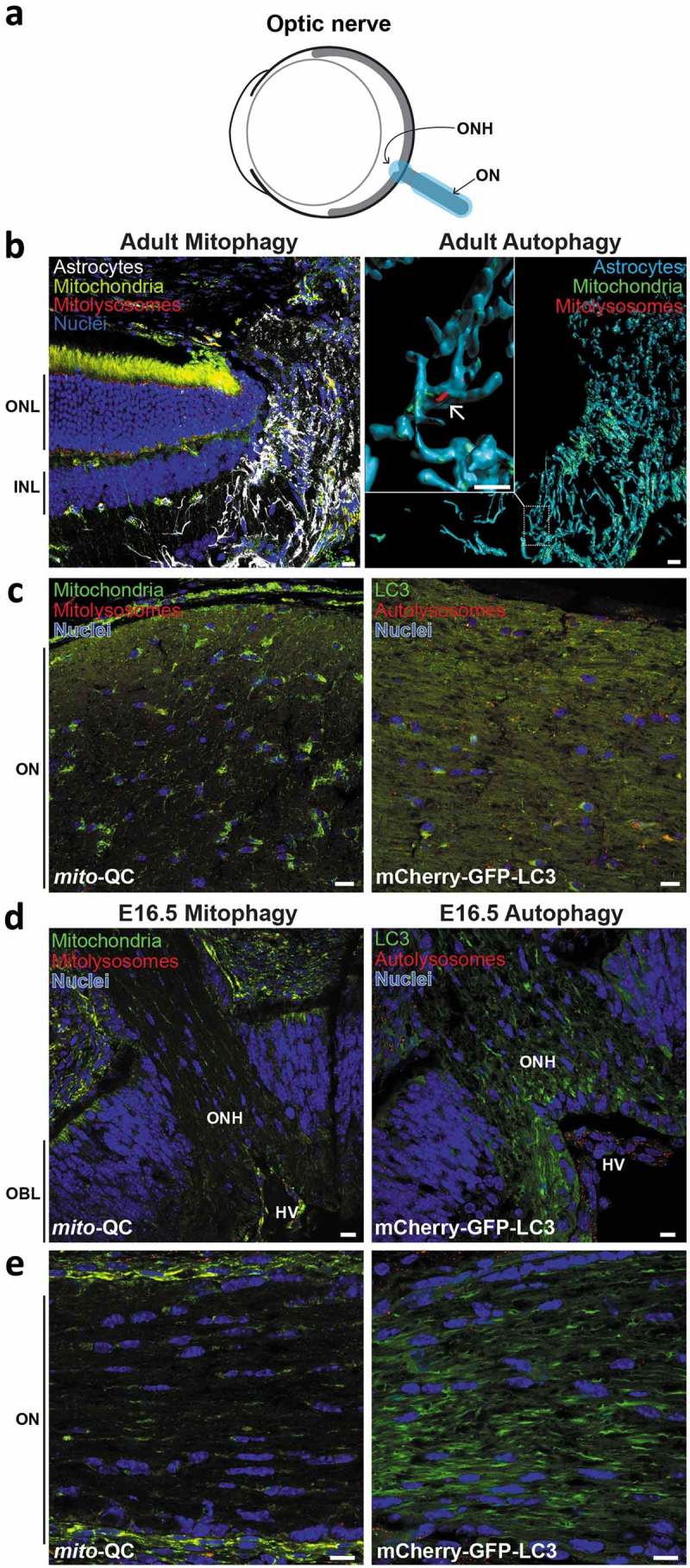
Minimal mitophagy and macroautophagy in the optic nerve. (a) Schematic of the mouse eye depicting anatomical location of the optic nerve in light blue. (b) Z-projection showing ONH of adult *mito*-QC eye, stained with anti-GFAP to mark astrocytes (white, left panel). Note retina on the left with outer nuclear layer (ONL) and inner nuclear layer (INL) marked. Right panel shows isosurface rendering of the ONH astrocytes (cyan) and boxed region is shown enlarged to the left. Mitolysosomes are sparse and the arrow indicates an example. (c) Optical section of ON from mito-QC (left panel) or mCherry-GFP-LC3 (right panel) eye. (d) Optical section of ONH from mito-QC (left panel) or mCherry-GFP-LC3 (right panel) E16.5 eyes. Outer neuroblast layer (OBL) of the developing retina is indicated. (e) Optical section of ON from mito-QC (left panel) or mCherry-GFP-LC3 (right panel) E15.5 eyes. Scale bars: 10 *μ*m.

Autophagy is often thought of as an intracellular catabolic process; however, recent studies have provoked a reassessment of this assumption. In particular, mitophagy has been proposed to be enriched at the optic nerve head (ONH) *in vivo*, by a process known as axonal trans-mitophagy [2,55]. During this process, damaged neuronal mitochondria are extruded from axonal evulsions within the optic nerve head, and are degraded by neighboring glial cells in a lysosome-dependent fashion [55]. When we assessed mitophagy at the neuro-retinal interface in *mito*-QC animals, depicted in Figure 6(a), we did not observe a high level of mitophagy (Figure 6(b). To determine if there are glial cells here, we stained with anti-GFAP (glial fibrillary acidic protein), which marks the astrocytes present in this region. On the whole, little mitophagy was seen in these ONH astrocytes, though there was clear evidence for mitophagy occurring in some of the cells (Figure 6(b)). Whether this constitutes an example of transmitophagy, or conventional mitophagy remains to be determined.

We also examined the optic nerve itself for mitophagy and general macroautophagy (Figure 6(c)). We observed almost no mitophagy and very little general autophagy here, but perhaps this is unsurprising given the axon-rich nature of this region and our previous observations of low axonal mitophagy [20]. This paucity of mitophagy and autophagy at the ONH and optic nerve was also present in E16.5 eyes (Figure 6(d,e)).

We also assessed mitophagy in the retinal pigment epithelium (RPE) in *mito*-QC mice. The RPE is the outermost layer of the retina and interfaces with the choroid membrane (Figure S3A). The RPE performs multiple critical functions that are essential for the visual cycle [56], and loss of autophagy in these cells leads to their degeneration [57]. Autophagy in the RPE has also been linked to age-related macular degeneration, highlighting its potential pathological importance in this region [58–60]. The RPE consists of a single layer of binucleated cells and by generating a 3D image from confocal sections, we were able to observe their unique hexagonal shape. These cells are rich in mitochondria, with a particular sub-plasmalemmal enrichment, and display a robust level of mitophagy *in vivo*, which can be clearly seen in isolated RPE (Figure S3C). It is interesting to note that these cells have been reported to undergo varying amounts of autophagy dependent on the day/night cycle and in particular undergo LC3-associated phagocytosis of shed photoreceptor outer segments [61]. While we previously detected no significant changes in retinal mitophagy at the end of the daily light or dark cycle [20], we were unable to reliable determine autophagy or mitophagy in the RPE *in situ* during this time course. We anticipate that future studies will address this.

## Discussion

To our knowledge, this exploratory study constitutes the first comparative *in vivo* landscape of basal mitophagy and macroautophagy in a mammalian organ system. Here we have focussed on the mammalian eye because numerous studies have linked aberrant mitophagy and autophagy to ocular pathology, yet little is known about the spatiotemporal nature of these processes within this organ. Additionally, we could not find any study that assessed the basal status of mitophagy with respect to the levels of total macroautophagy. Our work has demonstrated that macroautophagy is a significant process within the eye, occurring to varying degrees within all regions analyzed. In contrast in the adult, the level of mitophagy varied to a greater degree, and in comparing both models, mitophagy accounted for a much smaller fraction of the macroautophagy in the ciliary body and the lens in contrast to the cornea and especially the retina. Of course, the *mito*-QC model only measures the endpointof mitophagy, i.e. selective delivery of mitochondria to lysosomes, and the precise pathways at play that mark mitochondria for destruction across the various tissues of the eye remain to be elucidated. It is thus possible that some of the mitophagy observed occurs *via* a non-classical LC3-independent route. Regardless, in using *mito*-QC to monitor mitophagy and mCherry-GFP-LC3 mice to monitor macroautophagy, these data support the emerging notion that mitophagy is a highly context-dependent process *in vivo*, and not merely a consequence of stress or non-selective general autophagy.

Although beyond the scope of this resource paper, several interesting questions arise from our exploratory observations, relating both to selective autophagy and mitochondrial homeostasis. Notably, why does mitophagy occur at such a low rate in the lens or ciliary body, despite pronounced levels of general autophagy? These data highlight the selectivity of LC3-dependent macroautophagy, as while organellophagy may occur, mitochondria are seemingly spared. Other questions relate to the fundamental metabolic nature of tissues within the same organ. For example, why is there a high turnover of mitochondria specifically in the retinal ONL? Could the heightened cellular activity and neural nature of photoreception confer a greater metabolic stress or demand, which in turn accounts for differences in mitophagy between the ONL compared to other retinal cell types? Further questions also abound, especially with respect to selective autophagy *in vivo*. Does the enrichment of LC3-dependent macroautophagy in some tissues, and reciprocal lack of mitophagy in others, imply that autophagy in these structures is non-selective? Our discovery of hyaloid vascular mitophagy *in vivo* also extends our knowledge of mitochondrial turnover in endothelial networks and of general mammalian development. Future studies will be vital to determine the sequence of events pertaining to mitophagy and programmed cell death in these structures, and other vasculature in the eye.

In summary, this exploratory study constitutes the first comparative landscape of mammalian mitophagy and macroautophagy in the developing and mature eye *in vivo*. We anticipate our data will provide a fruitful resource for discovery-based and translational researchers to pursue much needed in-depth studies on ocular autophagy and its relationship to health and disease *in vivo*.

## Materials and methods

### Animals

*mito*-QC mice were generated as previously described [19]. Autophagy reporter mice (mCherry-GFP-MAP1LC3B, referred to as mCherry-GFP-LC3) were generated in the same way as *mito*-QC using targeted transgenesis by TaconicArtemis GmbH as described [20]. Mice were maintained on a C57BL6/J background. Genotyping was performed by diagnostic end-point PCR using genomic DNA isolated from tissue biopsy specimens. WT and KO alleles were detected using KOD Hot Start DNA polymerase (EMD Millipore, 71086) and manufacturer-recommended conditions. All animal studies and breeding were approved by the University of Dundee ethical review committee, and further subjected to approved study plans by the Named Veterinary Surgeon and Compliance Officer (Dr. Ngaire Dennison) and performed under a UK Home Office project license in accordance with the Animal Scientific Procedures Act of 1986.

### Histology & microscopy

Histology and microscopy were performed as previously described [19,20,45]. Briefly, mice were transcardially perfused with PBS (Gibco, 14190-094) and tissues were processed by immersion fixation in freshly prepared 3.7% formaldehyde (Sigma-Aldrich, P6148) at pH 7.0 in 0.2 M HEPES. For cryosectioning, eyes were cryoprotected in 30% (w:v) sucrose (VWR Chemicals, 27480.360) in PBS at 4°C before sectioning on a cryostat and counterstained with 1 µg/ml Hoechst (ThermoFisher Scientific, 62249).

For RPE isolation, the eyes were removed from the animal, and then the anterior eye portion was removed. The posterior eyecup was flattened after performing 4 incisions. The neural retina was separated from the RPE and the whole mount RPE was fixed for 16 h at 4ºC. DAPI (ThermoFisher Scientific, D1306) was used to counterstain the nuclei.

Vectashield (Vector Laboratories, H-1000) was used to mount tissue sections on slides before sealing with nail polish and a 1.5 glass coverslip.

Images were acquired using an LSM710 Multiphoton (Plan-Neofuar ×40 objective, NA 1.30; Plan Apochromat ×63 objective NA 1.4; Plan Apochromat ×20 objective, NA 0.8), or a LSM880 Airyscan microscope (ZEISS; Plan Apochromat ×63 objective, NA 1.4) and processed using ZEISS Zen Software/Adobe Photoshop or Imaris (Bitplane) for 3D Isosurface Rendering. Images were digitally altered within linear parameters, with minimal adjustments to levels and linear contrast applied to all images.

### Immunohistochemistry

Eye cryosections were re-fixed with formaldehyde 3.7% (w:v) in 200 mM HEPES buffer, pH 7.0 for 15 min, followed by 3 5-min PBS washes with agitation at room temperature. Retinal sections were permeabilized with 1% (v:v) Triton X-100 (Sigma-Aldrich, T8787), in PBS and then blocked for 1 h with BGT (3 mg/mL BSA [Roche, 10735108001], 0.25% Triton X-100, 100 mM Glycine [VWR Chemicals, 0167] in PBS). Primary antibodies were incubated in BGT overnight at 4ºC. Secondary antibodies were incubated for 1 h at room-temperature in BGT and darkness. Nuclei were stained with DAPI (1 μg/mL) and cryosections were mounted with Vectashield. Antibodies used were: CALB1 (Sigma-Aldrich, C2724), PRKCA (Sigma-Aldrich, P4334), SNCG (Abnova, H00006623-M01), AIF1 (Wako, 019-19741), ARR3 (EMD Millipore, AB15282), GLUL (EMD Millipore, MAB302), SAG (Santa Cruz Biotechnology, sc-166383,), and GFAP (Dako, Z0334).

### Data quantification

Data were quantified with Volocity 6.3 Image Analysis Software (PerkinElmer) using algorithms developed to analyze object overlap and count individual structures. For all analyses, we obtained images using uniform random sampling by an experimenter blind to all conditions. All images in each experimental group were processed as a batch using identical protocols. Images were first filtered to suppress noise (3x3 median filter) and a red/green intensity ratio channel was calculated. Similar analysis protocols were used for all eye regions. 1) The tissue was segmented using a fixed intensity threshold on the DAPI or green channel, followed by a fill holes operation and minimum size criterion. 2) To assess autophagy/mitophagy, objects were found in the red channel using mean intensity +n standard deviations (3 standard deviations for all tissues except the cornea, where the threshold was set at 2 standard deviations). Touching objects were separated using a guide size of 0.1 µm^2^. These objects were filtered according to a minimum red/green ratio value: 0.7 for all tissues except lens (1.0) and ciliary body (3.0). Differences in threshold values reflect the fact that expression levels and imaging settings were different between these eye regions. Finally, objects not overlapping with the tissue were excluded.

### Statistical analyses

For pairwise statistical analyses of mitophagy *vs*. macroautophagy comparisons, unpaired, two-tailed *t* tests with Welch’s correction were carried out. Statistics and graphs were generated using Prism 7 (GraphPad Software Inc.). All graphs are depicted as scatter plots with the number of data points representing an individual animal subject.

## Supplementary Material

Supplemental Material
