# Supplementary material for: A comparative map of macroautophagy and mitophagy in the vertebrate eye
Source: Autophagy. 2019 Feb 20;15(7):1296–308. doi: 10.1080/15548627.2019.1580509 (PMC6613837; doi:10.1080/15548627.2019.1580509)
Supplement: Supplemental Material [file kaup-15-07-1580509-s0001.zip › Supplementary information/Supplementary caption.docx]

**Figure S1.** Mitophagy and macroautophagy in embryonic hyaloid vasculature. (**A**) Schematic of the embryonic mouse eye depicting anatomical location of the hyaloid network in light blue. On the right is a low magnification optical section of E16.5 *mito*-QC lens with hyaloid vasculature (HV) indicated by dotted line. Scale bar: 100 *μ*m. (**B**) Optical section showing hyaloid vasculature from *mito*-QC (top panel) or mCherry-GFP-LC3 (bottom panel) E16.5 lens. Arrows show examples of mitolysosomes (top panel) and autolysosomes (bottom panel). Arrowheads mark autophagosomes. (**C**) Optical section and z-projection (**D**) from *mito*-QC perinatal eyes show mitophagy in perinatal hyaloid network situated next to the embryonic lens (EL) and spanning the vitreous chamber (VC). Boxed region of C is shown magnified below where the lumen (L) of the vessels can be clearly seen. Panels below D show 3D isosurface render of mitophagy and mitochondrial architecture in these structures. Arrows mark examples of mitolysosomes. Scale bars (B-D): 10 *μ*m.

**Figure S2.** Low level of retinal mitophagy outwith the outer nuclear layer. Retinal sections from *mito*-QC eyes were stained with various markers to identify the indicated cell types (in cyan). (**A**) Bipolar cells identified by anti-PRKCA/protein kinase Cα. (**B**) Amacrine cells, as well as horizontal cells, are identified by anti-CALB1/calbindin. (**C**) Retinal ganglion cells identified with SNCG/γ-synuclein. (**D**) Muller cells identified with anti-GLUL/glutamine synthetase. (**E**) Microglia identified with anti-AIF1/IBA1. Note lack of mitolysosomes in labelled cells. IS, inner segment; ONL, outer nuclear layer; OPL, outer plexiform layer; INL; inner nuclear layer; IPL, inner plexiform layer; GCL, ganglion cell layer Scale bars: 10 *μ*m.

**Figure S3.** Mitophagy in the retinal pigment epithelium. (**A**) Schematic of the eye depicting location of the retinal pigment epithelium in blue. (**B**) 3D image projection of the outer retinal region. Note hexagonal shape of retinal pigment epithelium (RPE) cells. OS, outer segments; IS, inner segments, ONL, outer nuclear layer. To the right is an optical section highlighting the single layer of RPE cells. (**C**) RPE flatmount displaying the hexagonal shape of RPE cells and sub-plasmalemmal mitochondria. Arrows mark examples of mitophagosomes. Scale bars: 10 *μ*m
