# Supplementary figures and images for: A comparative map of macroautophagy and mitophagy in the vertebrate eye
Source: Autophagy. 2019 Feb 20;15(7):1296–308. doi: 10.1080/15548627.2019.1580509 (PMC6613837; doi:10.1080/15548627.2019.1580509)

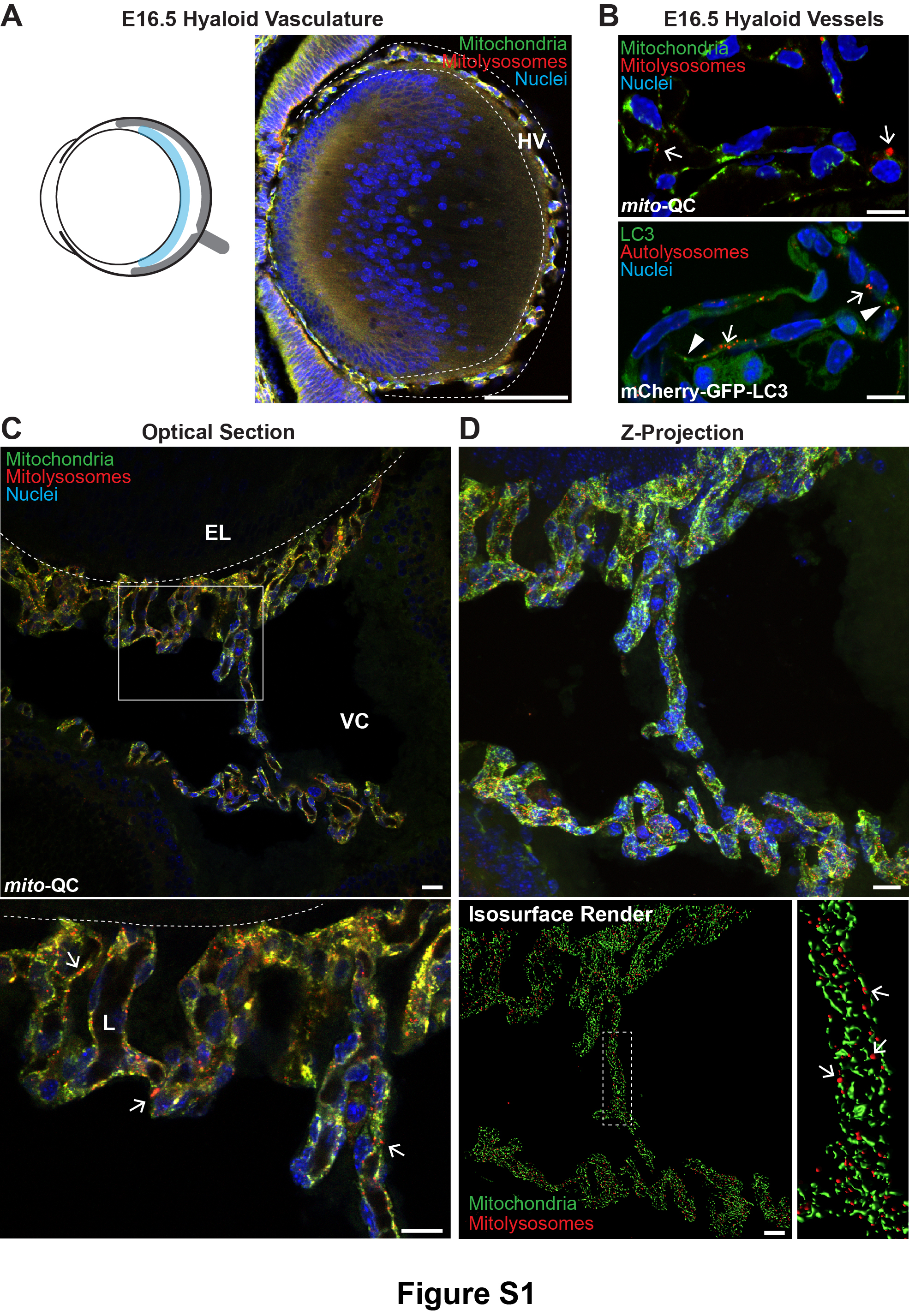

Supplement: Supplemental Material [file kaup-15-07-1580509-s0001.zip › Supplementary information/Fig-S1.jpg]

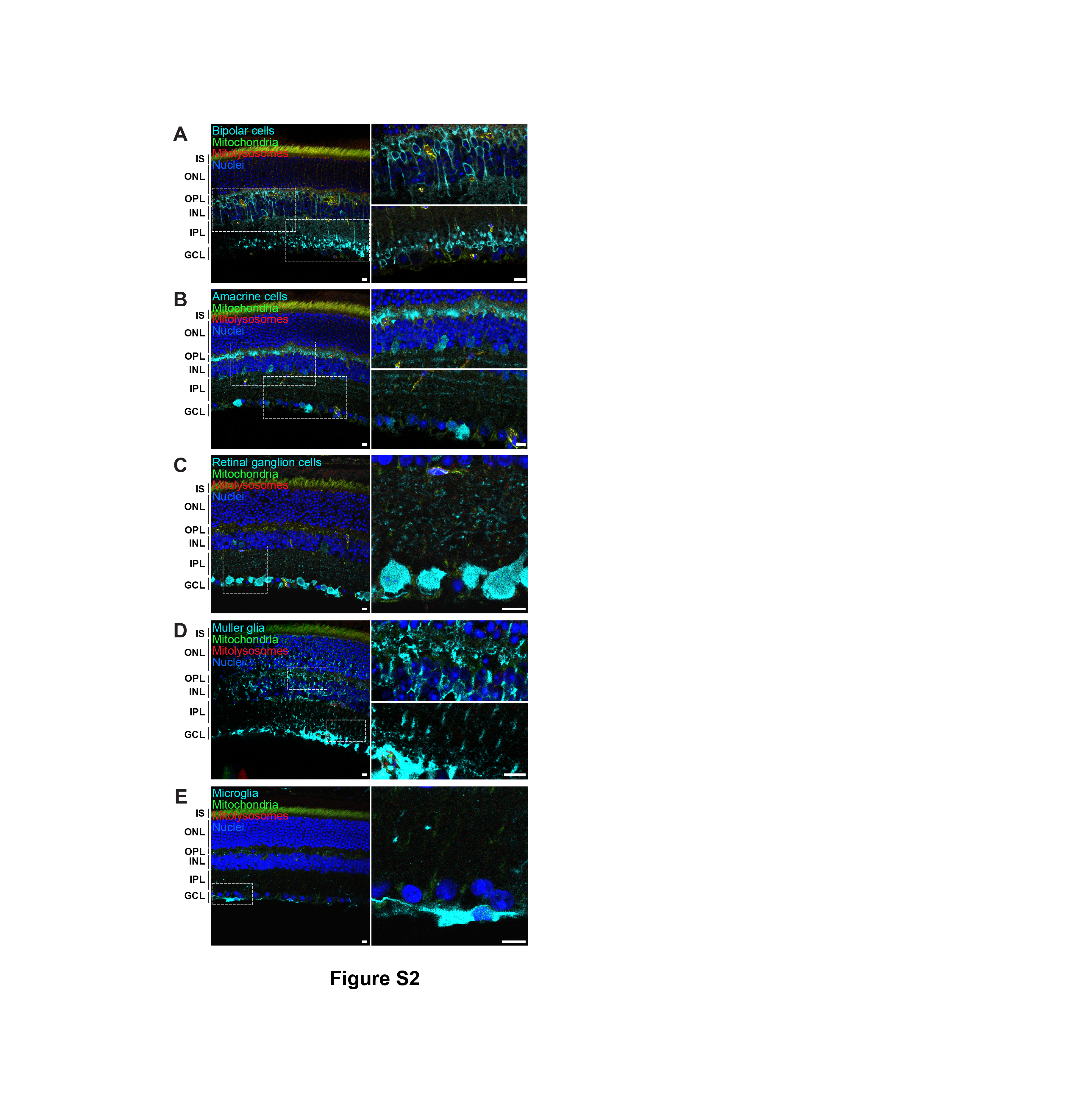

Supplement: Supplemental Material [file kaup-15-07-1580509-s0001.zip › Supplementary information/Fig-S2.jpg]

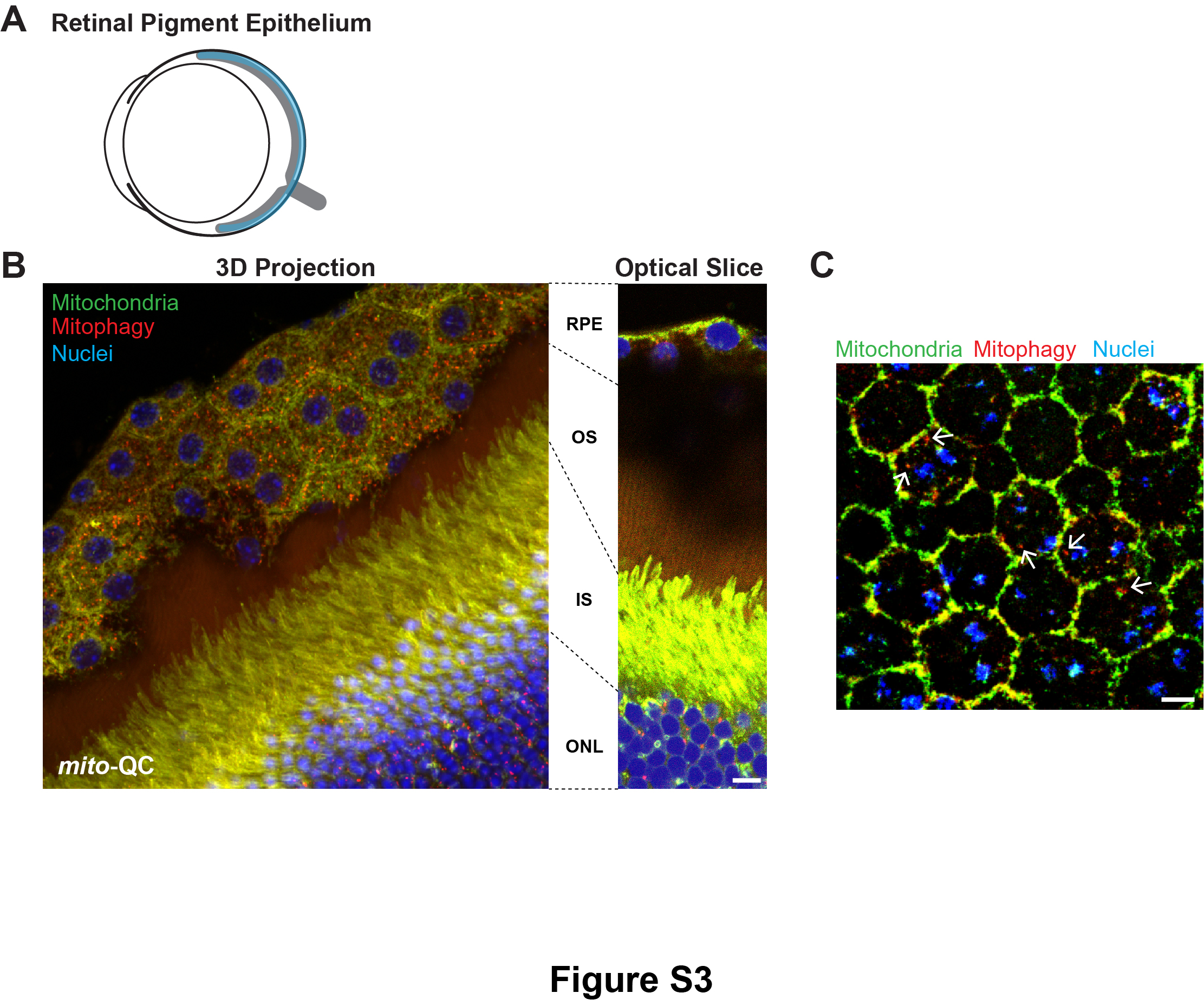

Supplement: Supplemental Material [file kaup-15-07-1580509-s0001.zip › Supplementary information/Fig-S3.jpg]
